# Upregulation of *Amy1* in the salivary glands of mice exposed to a lunar gravity environment using the multiple artificial gravity research system

**DOI:** 10.3389/fphys.2024.1417719

**Published:** 2024-06-26

**Authors:** Takehito Ouchi, Kyosuke Kono, Ryouichi Satou, Ryuya Kurashima, Koji Yamaguchi, Maki Kimura, Yoshiyuki Shibukawa

**Affiliations:** ^1^ Department of Physiology, Tokyo Dental College, Tokyo, Japan; ^2^ Department of Epidemiology and Public Health, Tokyo Dental College, Tokyo, Japan; ^3^ NeSTRA (Next-Generation Space System Technology Research Association), Yokohama, Japan

**Keywords:** lunar gravity, salivary gland, small G protein, amy1, microarray

## Abstract

**Introduction:** Space is a unique environment characterized by isolation from community life and exposure to circadian misalignment, microgravity, and space radiation. These multiple differences from those experienced on the earth may cause systemic and local tissue stress. Autonomic nerves, including sympathetic and parasympathetic nerves, regulate functions in multiple organs. Saliva is secreted from the salivary gland, which is regulated by autonomic nerves, and plays several important roles in the oral cavity and digestive processes. The balance of the autonomic nervous system in the seromucous glands, such as the submandibular glands, precisely controls serous and mucous saliva. Psychological stress, radiation damage, and other triggers can cause an imbalance in salivary secretion systems. A previous study reported that amylase is a stress marker in behavioral medicine and space flight crews; however, the detailed mechanisms underlying amylase regulation in the space environment are still unknown.

**Methods:** In this study, we aimed to elucidate how lunar gravity (1/6 *g*) changes mRNA expression patterns in the salivary gland. Using a multiple artificial gravity research system during space flight in the International Space Station, we studied the effects of two different gravitational levels, lunar and Earth gravity, on the submandibular glands of mice. All mice survived, returned to Earth from space, and their submandibular glands were collected 2 days after landing.

**Results:** We found that lunar gravity induced the expression of the salivary amylase gene *Amy1*; however, no increase in *Aqp5* and *Ano1*, which regulate water secretion, was observed. In addition, genes involved in the exocrine system, such as vesicle-associated membrane protein 8 (*Vamp8*) and small G proteins, including *Rap1* and *Rab* families, were upregulated under lunar gravity.

**Conclusion:** These results imply that lunar gravity upregulates salivary amylase secretion via *Rap*/*Rab* signaling and exocytosis via *Vamp8*. Our study highlights *Amy1* as a potential candidate marker for stress regulation in salivary glands in the lunar gravity environment.

## 1 Introduction

The transition from 1 *g* to the space environment changes not only gravity but also various other conditions such as the circadian rhythm (Otsuka et al., 2018) and exposure to cosmic rays (Zeitlin et al., 2013). It is important to elucidate the medical risks that humans face when living in a space environment, such as the oxidative stress caused by cosmic radiation (Overbey et al., 2019) and mechanical stress caused by microgravity (Dadwal et al., 2019). Artificial imitation of these environments may provide an understanding of gravitational physiology and determine how to avoid space stress.

A previous study showed that the gravitational threshold in the space environment provides a platform for exploring the mechanisms controlling the skeletal muscle response to alterations in gravity (Hayashi et al., 2023). Elucidation of the functional changes and molecular mechanisms involved may lead to the development of strategies to maintain human health during future long-term space travel. The musculoskeletal system and muscle movement controlled by somatic nerves have been studied previously in space and artificial gravitational environments (Juhl et al., 2021; Hayashi et al., 2023). Although studies on the heart rate and activity rhythms controlled by the autonomic nervous system in space are addressed (Blaber et al., 2004; Liu et al., 2015), research on the salivary glands, which are representative of local fluid balance regulation by the system, are limited. Saliva maintains the moist environment of oral tissues and is essential not only for feeding, mastication, and swallowing food but also for oral functions such as digestion, and articulation. In addition, saliva has antibacterial properties that help control the growth of harmful bacteria in the mouth, contributing to oral hygiene and prevention of dental issues such as cavities and gum disease. Saliva helps maintain pH balance in the mouth and neutralizes the acids produced by bacteria, thereby reducing the risk of tooth decay and erosion. It plays a role in taste perception by dissolving food particles and allowing them to interact with the taste buds in the tongue. Therefore, saliva is a multifunctional fluid that contributes significantly to oral and digestive health. Insufficient saliva production, known as dry mouth or xerostomia, can lead to various oral health problems and difficulties chewing, swallowing, and speaking. However, little is known about the regulatory mechanisms of salivary secretion during spaceflights.

In this study, we collected submandibular glands from mice exposed to artificial lunar gravity.

Among the major salivary glands, the large amount of saliva secretion comes from the submandibular gland. Therefore, we thought that changes in salivary gland mRNA due to environmental changes might be more clearly observed in the submandibular gland compared to other salivary glands. In addition, the submandibular gland is a mixed gland, and we considered that it would be a good model to observe the control mechanism and balance of protein secretion and water secretion by the sympathetic and parasympathetic nerves, i.e., under autonomic regulation.

Artificial lunar gravity was developed using multiple artificial gravity research systems (MARS) that use a centrifuge to create artificial gravity, enabling comparative studies between the effects of gravity and microgravity in space (Shimbo et al., 2016; Shiba et al., 2017; Okada et al., 2021). This system has contributed to analysis of sulfur metabolomic and transcriptomic changes in the liver (Kurosawa et al., 2021), metabolic effects in adipose tissues (Suzuki et al., 2020; Uruno et al., 2021), blood parameters changes (Shimizu et al., 2023), bone mineralization, blood pressure, and lipid metabolism in mouse kidneys (Suzuki et al., 2022) during space travel. This study investigated the basic foundations for oral clinical medicine under lunar gravity. We sought to understand whether lunar gravity (1/6 *g*) influence the mRNA expression in salivary glands during space flight. Using the developed MARS, two independent missions (MHU-4 and MHU-5) were conducted in the space. Using the MARS system, we analyzed the mRNA expression of the whole submandibular gland tissue of mice raised under lunar (1/6 *g*) and Earth gravity (1 *g*).

## 2 Materials and methods

### 2.1 Animals

C57BL/6 J male mice (Stock #000664) obtained from Jackson Laboratories (Bar Harbor, ME, United States) and Charles River Laboratories (Wilmington, MA, United States) were used for the MHU-4 and -5 missions. All experiments were approved by the Institutional Animal Care and Use Committee of the Japan Aerospace Exploration Agency (JAXA) (Protocol Number: No. 018-011D for MHU-4 and No. 018-036D for MHU-5), Explora Biolabs (Study Number: No. EB19-003 for MHU-4 and No. SP19-003 for MHU-5), and the National Aeronautics and Space Administration (NASA) (Protocol Number: No. FLT-18-118 for MHU-4 and No. JAXA MHU-5/FLT-19-121 for MHU-5). All experiments were conducted in accordance with the guidelines and applicable laws of Japan and the United States.

### 2.2 MHU missions

The ground control group (GC) comprised of six age-matched (9-week-old) male C57BL/6 J mice per mission, maintained on Earth under the same conditions as those aboard the International Space Station (ISS). A detailed description of the space flight experiments has been previously reported (Hayashi et al., 2023).

In the MHU-4 mission, six 9-week-old male C57BL/6 J mice in a transportation cage unit were launched aboard SpX-17 on 7 May 2019, from the NASA Kennedy Space Center and were then transported to the ISS. The MHU-4 partial gravity (PG) group was maintained in an artificial gravity environment of 1/6 *g* on the bottom floor of the habitat cage unit by centrifugation at 31 rpm in MARS using a short-radius (15 cm) centrifuge (MARS-S).

In the MHU-5 mission, six 9-week-old male C57BL/6 J mice in a transportation cage unit were launched aboard SpX-20 on 10 March 2020, from the NASA Kennedy Space Center and were then transported to the ISS. The MHU-5_PG group was maintained in an artificial gravity environment of 1/6 *g* on the bottom floor of the habitat cage unit by centrifugation at 21 rpm in MARS using a long-radius (35 cm) centrifuge (MARS-L).

The lighting in the mouse habitat cage considered the circadian rhythm and used a 12-h cycle according to the ISS and GMT (standard) time, with a light period of 7:00 to 19:00 and a dark period from 19:00 to 7:00 the next day.

Cage unit was equipped with a water dispenser and a feeder that can automatically supply water and food. The crew only needed to periodically refill the water and replenish the food, reducing the workload of the crew. The water dispenser was based on a balloon for injecting medicines, and drinking water was supplied by pressurizing the water using the elasticity of the balloon. The amount of water in the balloon was able to be detected by a sensor when it was full and when a certain amount of water had been consumed from full. For feeding, a spring was used to push out integrally molded food to the feeding surface. Food needed to be replenished once a week, but the food in this device was in cartridge form, making it easy for the crew to replace the cartridges.

All the mice from each mission were sacrificed on the second day after returning to the Earth.

### 2.3 Sample collection and preparation

Submandibular glands were fixed with 4% paraformaldehyde (Wako, Osaka, Japan) for 1–2 d at 4 °C before replacing the paraformaldehyde with phosphate-buffered saline (PBS). The MHU-4 mission samples were processed in the United States in June 2019, and the MHU-5 mission samples were processed in the United States in April 2020. After approximately 40 d, the samples were treated with different concentrations of methanol, 25%, 50%, 75%, and 100%, for 30 min each and then maintained in 100% methanol. The MHU-4 mission samples were processed in Japan in July 2019, and the MHU-5 mission samples were processed in Japan in May 2020.

Samples from the ground control experiments were treated using the same method as those from the space experiments. The MHU-4 mission, samples were processed in December 2019; and the MHU-5 samples were processed in October 2020.

### 2.4 Total RNA isolation

Total RNA from whole submandibular gland in each group (the MHU-4 GC, the MHU-4 PG, the MHU-5 GC, the MHU-5 PG); n = 1 for each was isolated from the cells using the RNeasy Mini Kit (QIAGEN, Germany), according to the manufacturer’s instructions. RNA samples were quantified using a Nanodrop ND-1000 spectrophotometer (Thermo Fisher Scientific, Waltham, MA, United States), and their quality was confirmed using a 2,200 TapeStation (Agilent Technologies, Santa Clara, CA, United States).

### 2.5 Gene expression microarrays

The total RNA from whole submandibular gland in each group was amplified using GeneChip^®^ WT Pico Kit (Thermo Fisher Scientific) and hybridized to the Clariom™ S Assay, mouse (Thermo Fisher Scientific), according to the manufacturer’s instructions. All hybridized microarrays were scanned using an Affymetrix scanner (Thermo Fisher Scientific). Relative hybridization intensities and background hybridization values were calculated using Affymetrix Expression Console™. Gene set enrichment analysis (GSEA) was conducted according to methods previously described (Mootha et al., 2003; Subramanian et al., 2005).

### 2.6 Data analysis and filter criteria

The raw signal intensities of all samples were normalized by a quantile algorithm using Affymetrix^®^ Power Tools version 1.15.0 software. To identify up- or downregulated genes, we calculated Z-scores [Z] and ratios (non-log scaled fold-change) from the normalized signal intensities of each probe for comparison between the control and experimental samples. The criteria for upregulated genes was Z-score ≥2.0 and ratio ≥1.5-fold and for downregulated genes was Z-score ≤ −2.0 and ratio ≤0.66. Since there were no replicate samples in this analysis, it was impossible to evaluate the data using *p*-values. We used intensity-based Z-scores and fold changes to extract differentially expressed mRNAs. This is expected to reduce noise compared to extracting variable mRNAs based only on fold change values (Quackenbush, 2002).

### 2.7 Immunofluorescence analysis

We conducted immunofluorescence staining by using the whole submandibular gland in each group (the MHU-4 GC, the MHU-4 PG, the MHU-5 GC, the MHU-5 PG); n = 1 for each. The fixed submandibular glands were embedded in paraffin, and 2 μm sections were prepared using a microtome (Leica Biosystems, Wetzlar, Germany). The sections were deparaffinized by immersing in xylene (Wako) for 10 min three times. The sections were then rehydrated using sequential immersion in 100%, 95%, and 70% ethanol for 10 min. After ethanol treatment, the sections were washed three times with PBS. For immunofluorescence analysis, the samples were permeabilized with Triton X-100 (Sigma Aldrich, St. Louis, MO, United States) for 5 min before incubating with blocking buffer (Nacalai Tesque, Kyoto, Japan) at room temperature for 15 min. To evaluate the protein expression patterns, the samples were then incubated overnight at 4 °C with the following primary antibodies: mouse monoclonal anti-Amylase (sc-46657; 1:200; Santa Cruz Biotechnology, Inc., Dallas, TX, United States), rabbit polyclonal anti-Aquaporin5 (ab53212; 1:200; Abcam, Cambridge, United Kingdom), mouse monoclonal anti-Rap1 (sc-398755; 1:200; Santa Cruz Biotechnology, Inc.), mouse monoclonal anti-Rab2a (67501-1-Ig; 1:200; Proteintech, Rosemont, IL, United States), rabbit polyclonal anti-Rab10 (11808-1-AP; 1:200; Proteintech), rabbit polyclonal anti-Rab27b (13412-1-AP; 1:200; Proteintech), and rabbit polyclonal anti-Vamp8 (15546-1-AP; 1:200; Proteintech).

After washing with PBS, the following secondary antibodies were then incubated for 1 h at room temperature: Alexa Fluor^®^ 568 donkey anti-mouse (#A10037; Thermo Fisher Scientific), Alexa Fluor^®^ 488 donkey anti-rabbit (#A21206, Thermo Fisher Scientific). After washing with PBS, the stained samples were mounted in a mounting reagent with 4,6-diamidino-2-phenylindole (ab104139; Abcam) and analyzed using a fluorescence microscope (X710, X800; Keyence, Osaka, Japan).

## 3 Results

**FIGURE 1:**
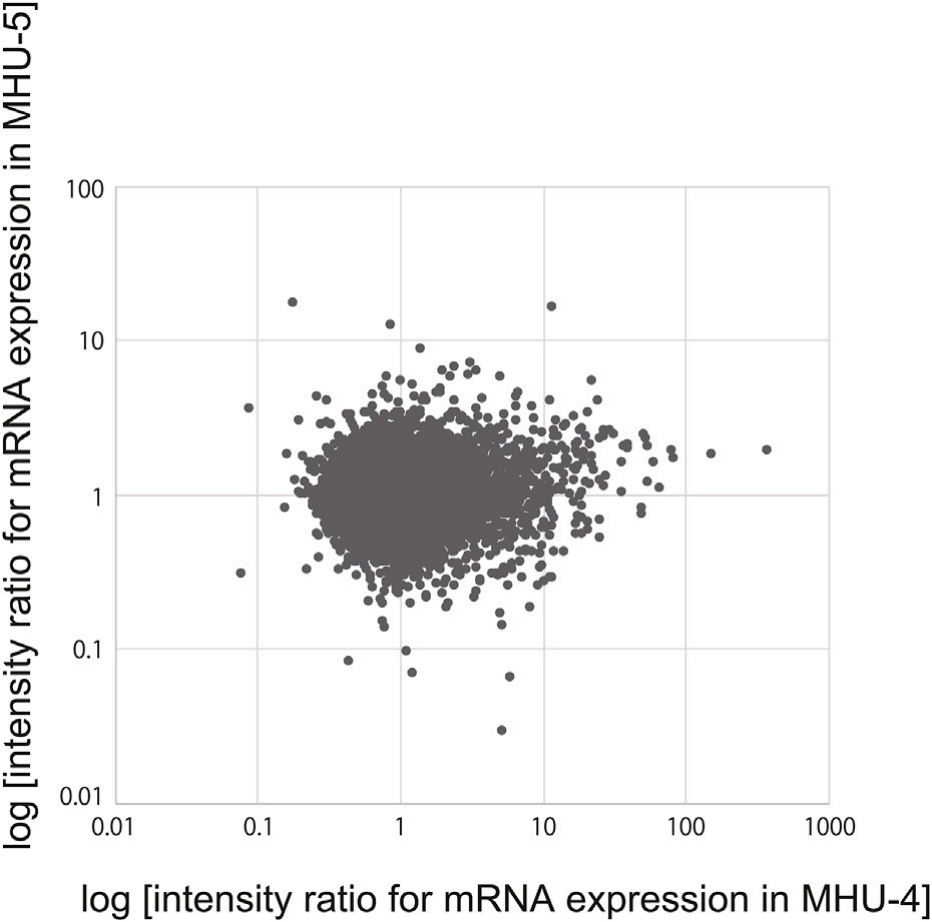
No correlation of salivary gene expression was observed between the MHU-4 and -5 missions. Microarray analysis revealed no correlation between changes in the mRNA expression of 22,206 genes (correlation coefficient: 0.077052) between the MHU-4 and -5 missions.

Microarray analysis to examine the changes in mRNA expression of the 22,206 genes found no correlation (correlation coefficient: 0.077052) between the submandibular gland samples obtained from the MHU-4 and -5 missions, even though both missions were conducted under the same lunar surface gravity environments (Figure 1). This may be due to differences in the centrifugal gradient caused by the radius of each MARS and circadian fluctuation between the time points for collecting samples in two missions.

**FIGURE 2:**
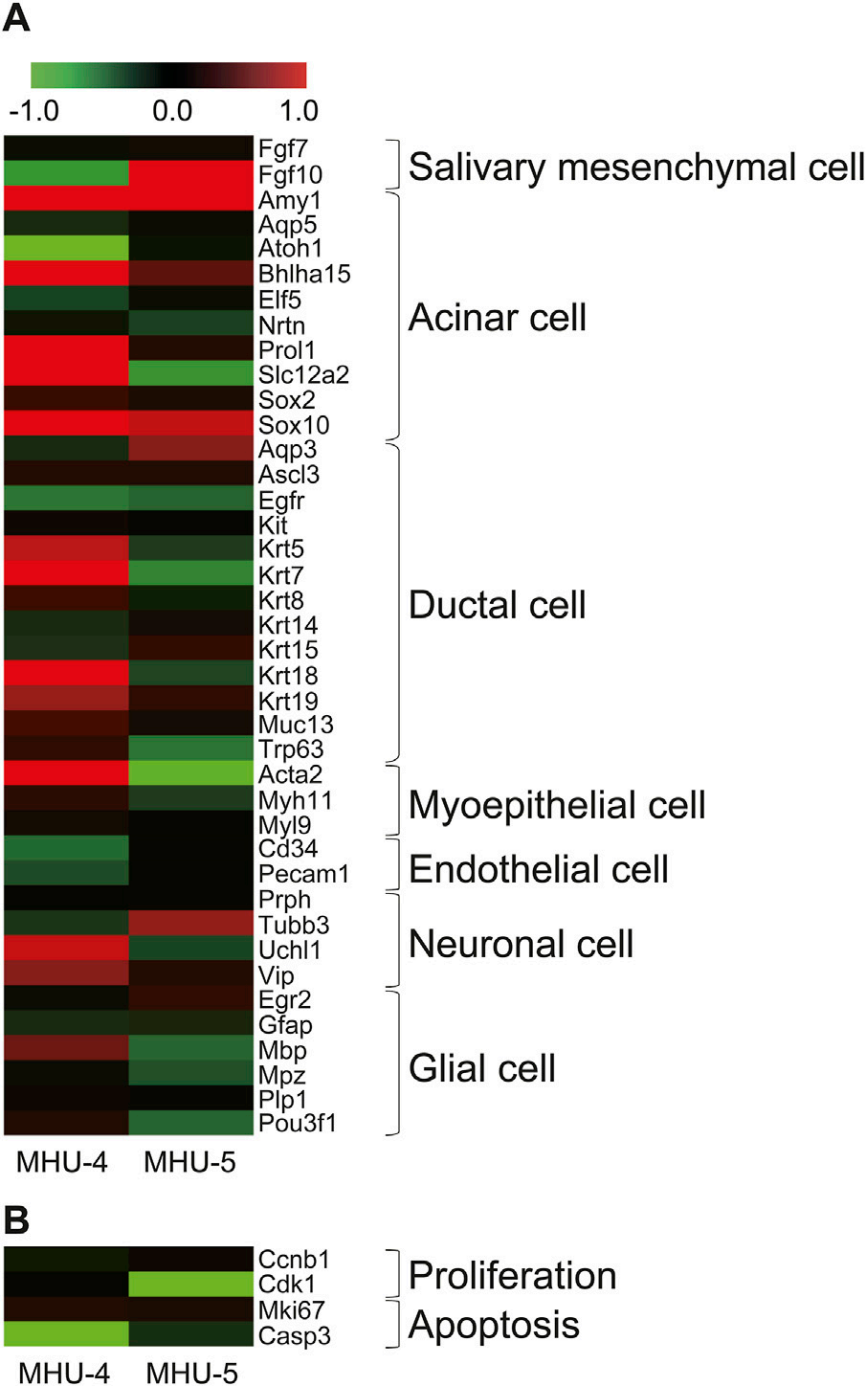
Increases of gene expression in acinar cells in lunar gravity environment. **(A)** Heat map shows representative mRNAs, encoding marker protein for the cells comprising submandibular gland. The map represents the ratio calculated from the signal intensity of 1/6 *g*–1 *g.* Microarray analysis revealed that the expression increases with significance were more seen in acinar cells. Gene expression increases were more observed in MHU-4 compared with those in MHU-5. **(B)** Genes relating to cell proliferation and apoptosis showed no upregulation. Red in heat map indicates upregulated mRNA at 1/6 *g* and green in heat map indicates downregulated mRNA at 1/6 *g* compared to the 1 *g* group.

We then investigated the cells comprising the submandibular glands and surrounding cell populations, based on specific marker, to identify which cell components showed specific mRNA expression changes in the lunar gravity environment in MHU-4 and -5. The heat map in Figure 2A shows signal intensity ratio of mRNA expression in samples at 1 *g* and 1/6 *g* environment in both MHU-4 and MHU-5 missions. Red indicates upregulated genes at 1/6 *g* and green indicates downregulated genes at 1/6 *g* compared to the 1 *g* group. Microarray analysis of whole submandibular glands samples from both MHU-4 and -5 revealed that the overall variation in mRNA expression was more pronounced in the MHU-4 mission than in the MHU-5 mission. Expression of mRNA relating the acinar cells, characterized by *Amy1*, *Bhlha15*, *Sox10* were more pronounced than those from other cell populations (Figure 2A). The expression of mRNA related to proliferation and apoptosis did not significantly increase at 1/6 *g* compared to the 1 *g* group (Figure 2B). Therefore, we focused on mature acinar cells that have physiological functions such as protein and water secretion.

**FIGURE 3:**
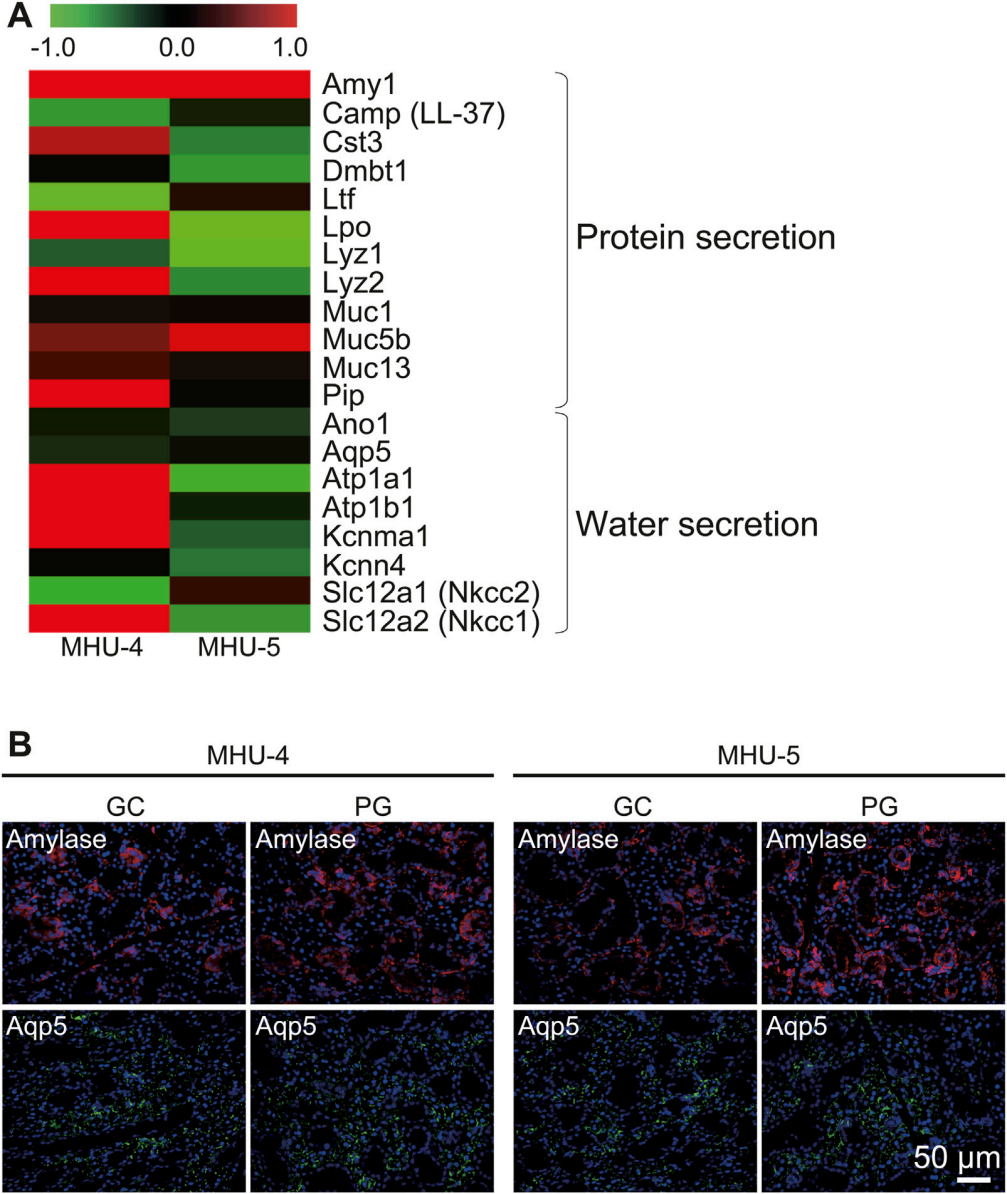
Upregulation of salivary *Amy1* in 1/6 *g* lunar gravity environment in both MHU-4 and MHU-5 missions. **(A)** Heat map showing the ratio calculated from the signal intensity of 1/6 *g*–1 *g.* Microarray analysis revealed that salivary protein gene *Amy1* was upregulated in 1/6 *g* lunar gravity in both MHU-4 and -5 missions. No significant increases of *Aqp5* were observed. Red in heat map indicates upregulated mRNA at 1/6 *g* and green in heat map indicates downregulated mRNA at 1/6 *g* compared to the 1 *g* group. **(B)** Immunofluorescence staining confirmed the increased expression of amylase in 1/6 *g* lunar gravity in both MHU-4 and -5 but no increase of Aqp5 even though the salivary cells were highly immunopositive for Aqp5. Scale bar: 50 μm.

*Amy1* encodes salivary amylase secreted by acinar cells, which is an organic component involved in serous saliva. *Amy1* showed significant upregulation in the samples in the 1/6 *g* environment during both MHU missions (Figure 3A). In contrast, *Aqp5*, which is involved in water secretion in acinar cells, was highly expressed (data not shown), but no significant changes in the mRNA expression were observed between lunar gravity and ground controls in either mission (Figure 3A). Immunofluorescence staining showed that the amylase protein was expressed at a higher level in lunar gravity than in the ground control, which was consistent with the microarray results. Aqp5 protein expression was also consistent with the microarray data (Figure 3B).

**FIGURE 4:**
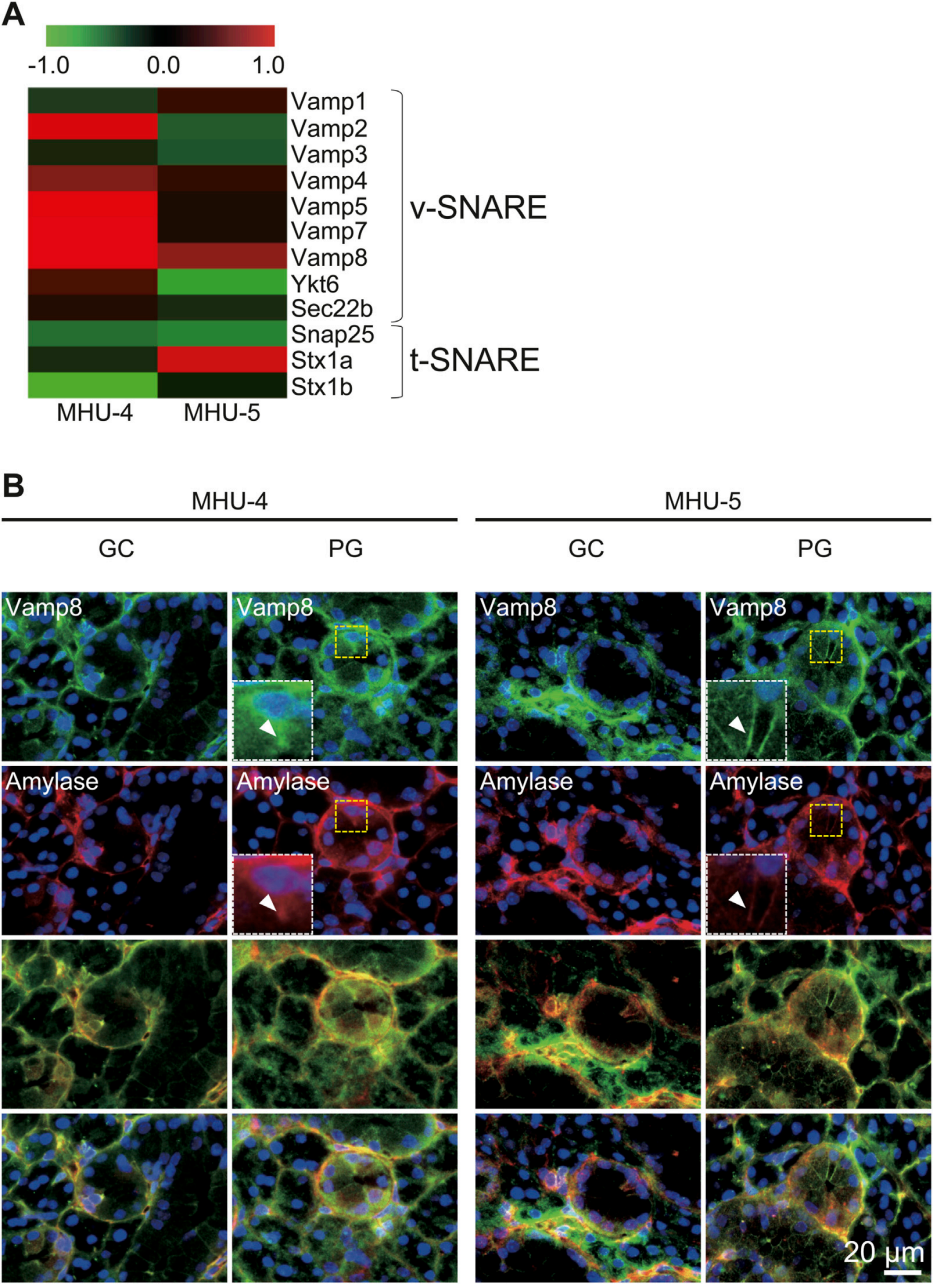
Upregulation of *Vamp8* in the lunar gravity group in both MHU-4 and MHU-5. **(A)** Heat map showing the ratio calculated from the signal intensity of 1/6 *g*–1 *g.* Microarray analysis revealed changes in expression of the v-SNARE gene, *Vamp8.* Other v-SNARE genes such as *Vamp2*, *5*, and *7* were upregulated at 1/6 *g* compared to the 1 *g* in MHU-4. Upregulation of the t-SNARE gene in lunar gravity was not observed. Red in heat map indicates upregulated mRNA at 1/6 *g* and green in heat map indicates downregulated mRNA at 1/6 *g* compared to the 1 *g* group. **(B)** Immunofluorescence staining confirmed the co-localization of amylase and Vamp8 in acinar cells. Co-localized expression of amylase and Vamp8 was higher in lunar gravity compared with ground control for MHU-4 and MHU-5 missions. Insets (white dotted rectangles) were enlarged from the yellow dotted rectangles. Arrowheads indicate strong expression of Vamp8 protein and amylase at apical side of cell membrane in acinar cells. Scale bar: 20 μm.

We further examined signal intensity ratio of mRNA for membrane proteins with cytosolic coiled-coil domains, termed SNAREs, which are found in vesicles (v-SNAREs) and organelles (t-SNAREs) in whole submandibular glands. Vesicle-associated membrane protein 8 (*Vamp8*) is a v-SNARE gene involved in protein vesicle secretion mainly by acinar cells and was highly expressed under lunar gravity in both MHU missions; however, there was no increase in t-SNARE (Figure 4A). Based on these results, it was assumed that amylase secretion was promoted through Vamp8 in the 1/6 *g* lunar gravity environment compared to 1 *g*. In samples from the both MHU-4 and MHU-5 missions, immunostaining revealed that amylase and Vamp8 protein expression was co-localized within acinar cells. We also observed strong expression of Vamp8 protein at apical side of cell membrane in acinar cells at 1/6 *g* environment, implying enhancement of vesicle-membrane docking to secrete salivary proteins (Figure 4B).

**FIGURE 5:**
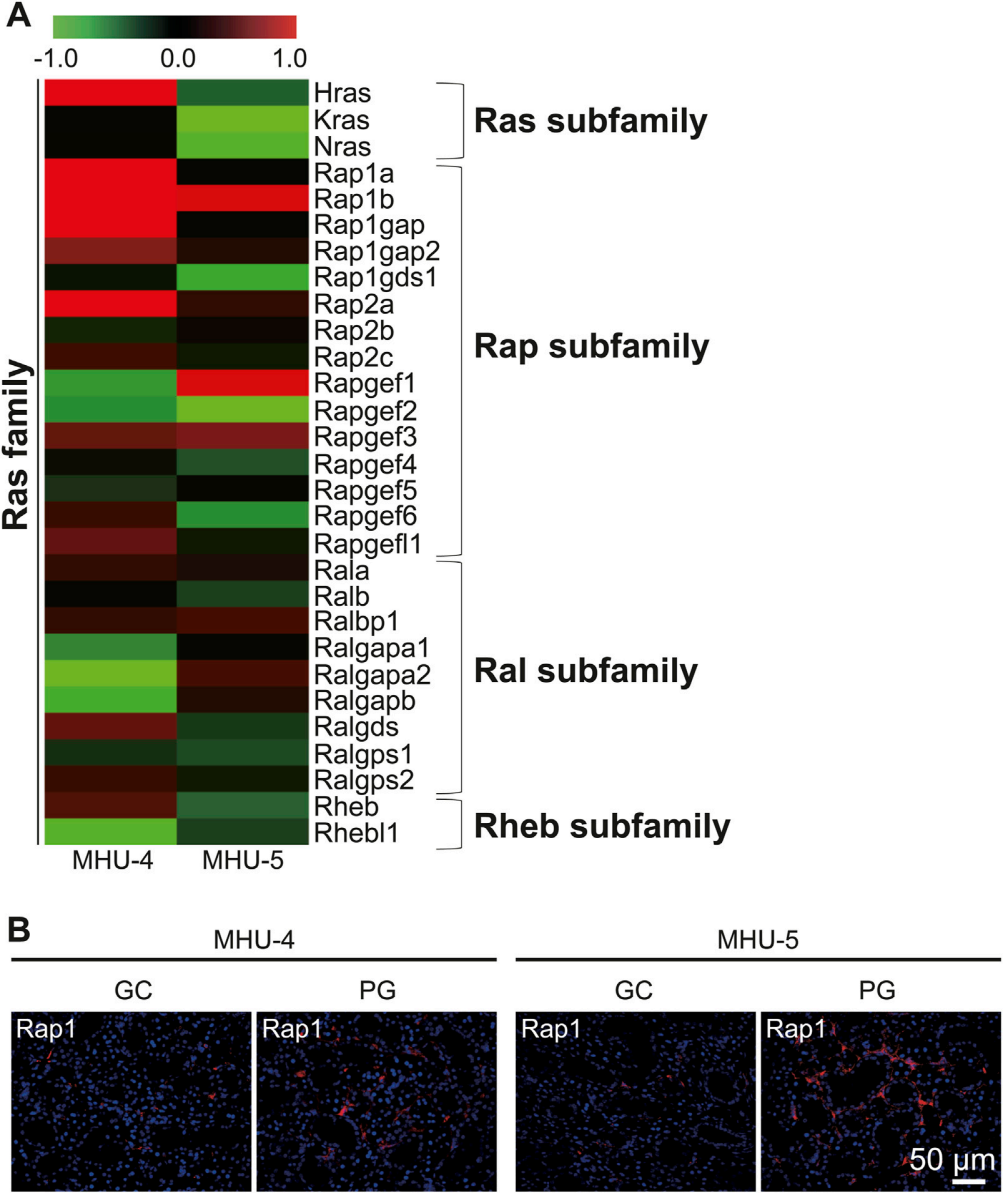
Upregulation of *Rap1* in lunar gravity in both MHU-4 and MHU-5. **(A)** Heat map showing the ratio calculated from the signal intensity of 1/6 *g*–1 *g.* Microarray analysis revealed changes in expression of *Rap1* in 1/6 *g* in both MHU-4 and MHU-5. Red in heat map indicates upregulated mRNA at 1/6 *g* and green in heat map indicates downregulated mRNA at 1/6 *g* compared to the 1 *g* group. **(B)** Immunofluorescence staining confirmed the upregulation of Rap1 in lunar gravity compared with ground control for both MHU-4 and MHU-5 missions. Scale bar: 50 μm.

**FIGURE 6:**
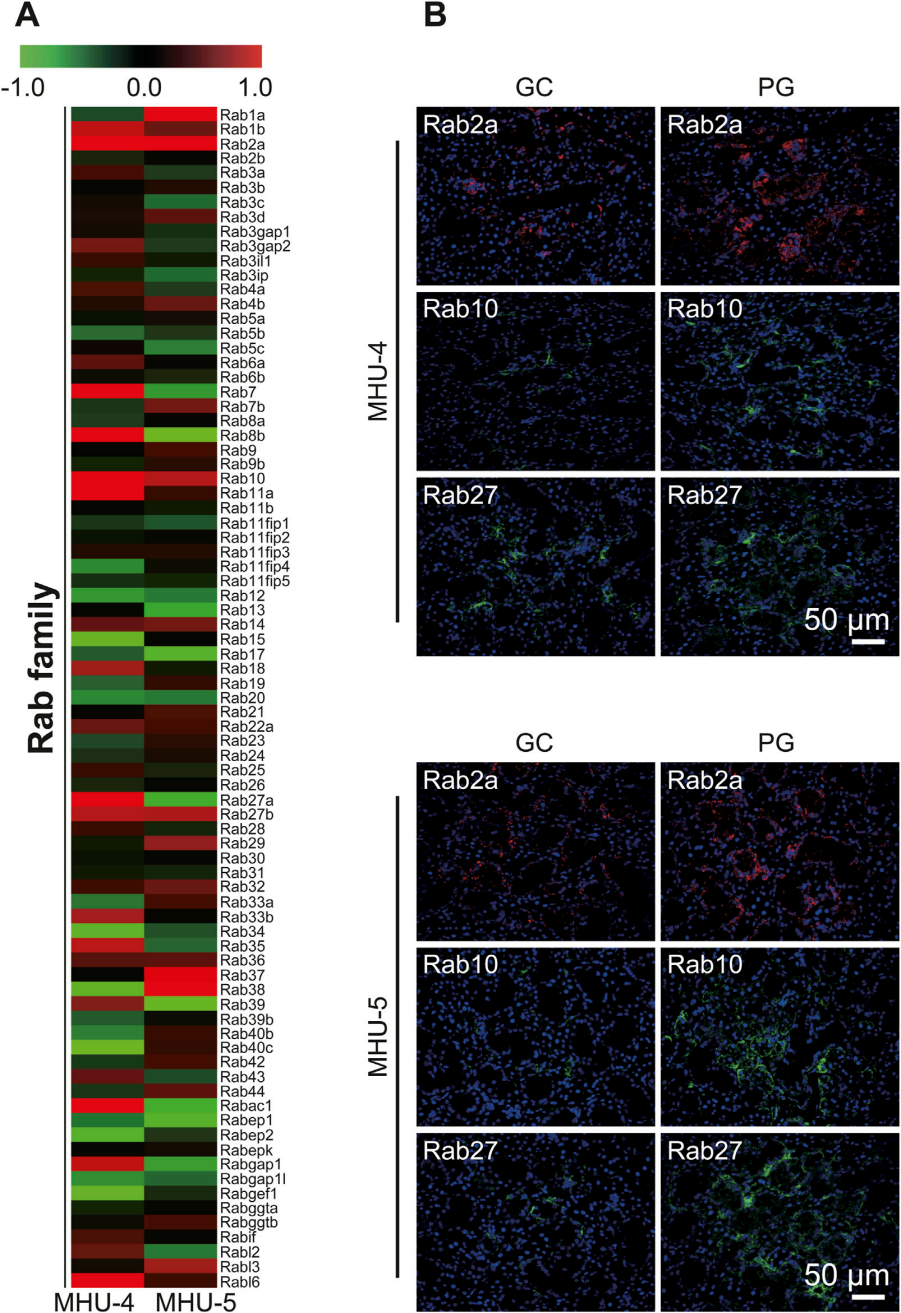
Upregulation of *Rab2a*, *Rab10*, and *Rab27* in lunar gravity in both MHU-4 and MHU-5. **(A)** Heat map showing the ratio calculated from the signal intensity of 1/6 *g*–1 *g.* Microarray analysis revealed that changes in the expression *Rab2a*, *Rab10*, and *Rab27* in 1/6 *g* in both MHU-4 and MHU-5. Red in heat map indicates upregulated mRNA at 1/6 *g* and green in heat map indicates downregulated mRNA at 1/6 *g* compared to the 1 *g* group. **(B)** Immunofluorescence staining confirmed the upregulation of Rab2a, Rab10, and Rab27 in lunar gravity compared with ground control for both MHU-4 and MHU-5 missions. Scale bar: 50 μm.

**FIGURE 7:**
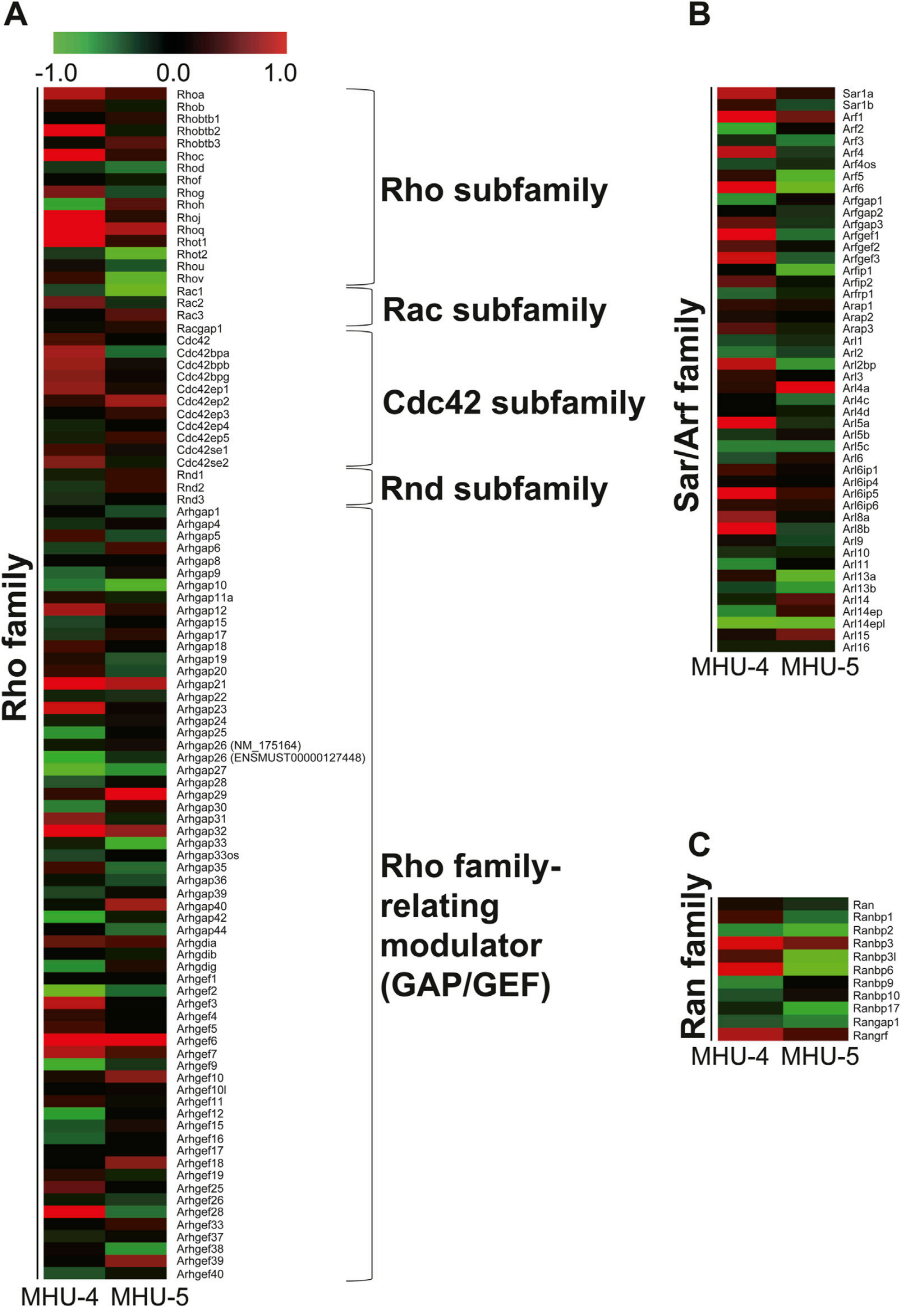
No common upregulation of *Rho*, *Sar/Arf*, and *Ran* family genes was observed in the lunar gravity groups of the MHU-4 and MHU-5 missions. **(A–C)** Heat map showing the ratio calculated from the signal intensity on small G protein, *Rho*, *Sar/Arf*, and *Ran* family of 1/6 *g*–1 *g*. Microarray analysis revealed no common upregulation of *Rho*, *Sar/Arf*, and *Ran* family except for *GEF*/*GAP* in lunar gravity for both MHU-4 and MHU-5 missions. Red in heat map indicates upregulated mRNA at 1/6 *g* and green in heat map indicates downregulated mRNA at 1/6 *g* compared to the 1 *g* group.

Microarray analysis showed an increase in the mRNA expression of *Rap1b*, a subfamily of the *Ras* family in the 1/6 *g* lunar gravity of both MHU-4 and -5 groups (Figure 5A). The immunofluorescence staining results showed the upregulation of Rap1 protein, which were similar with the microarray data (Figure 5B). Increases of mRNA expression in samples from PG in both MHU-4 and MHU-5 missions were also observed for *Rab1*, *Rab2a*, *Rab10*, and *Rab27*, which are members of the *Rab* family (Figure 6A). Immunofluorescence staining showed upregulation of Rab2a, Rab10, and Rab27 protein which were similar with the microarray data (Figure 6B). Upregulation of some mRNAs from the *Rho* (Figure 7A), *Sar*/*Arf* (Figure 7B), and *Ran* (Figure 7C) families was observed at 1/6 *g* samples but did not show common expression patterns between MHU-4 and -5 missions, except for GEF/GAP (Figures 7A–C).

**FIGURE 8:**
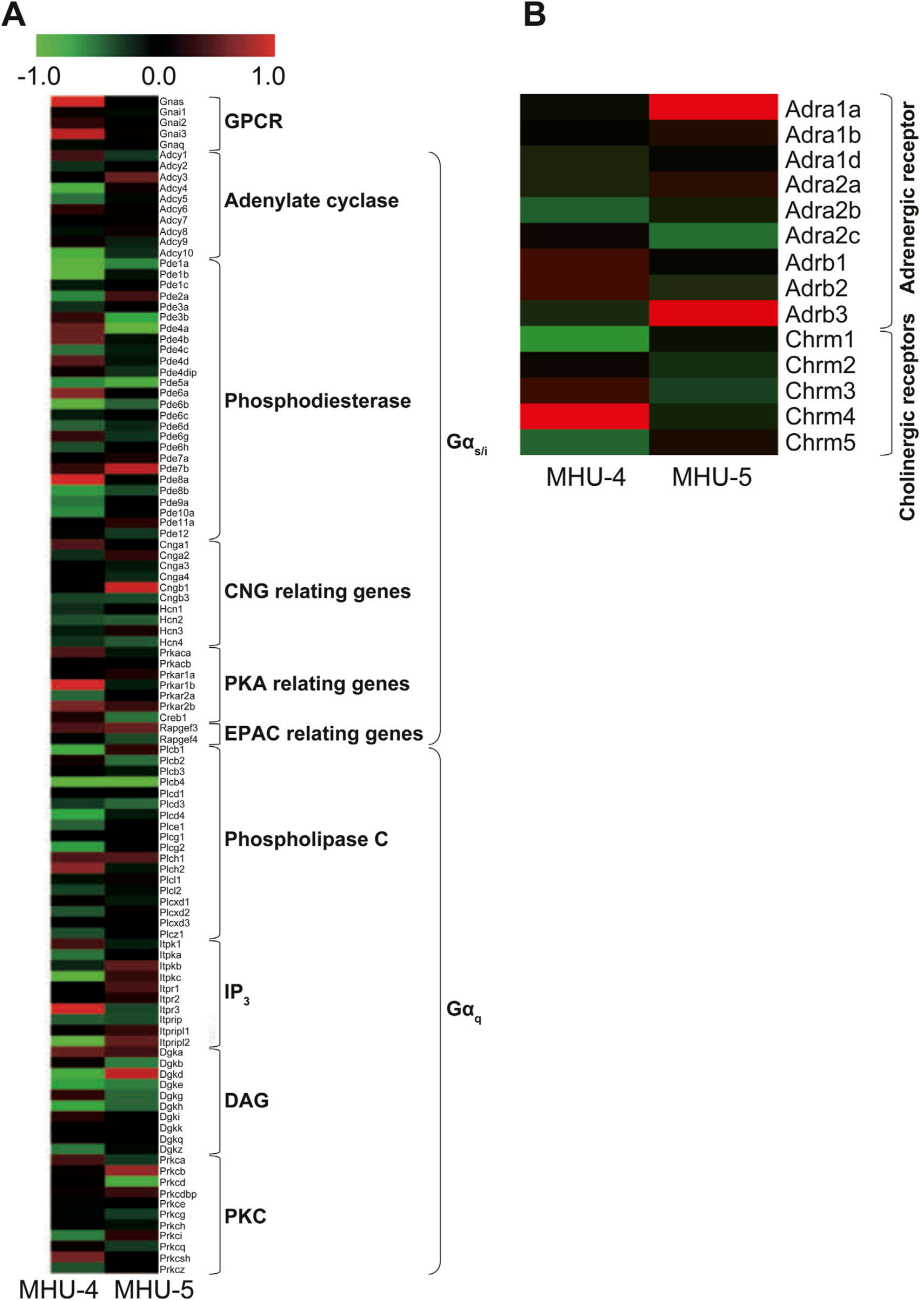
No common upregulation of GPCR, including G_s_, G_i_, or G_q_ proteins involved in salivary secretion, was observed. **(A)** Heat map showing the ratio calculated from the signal intensity of 1/6 *g*–1 *g.* Microarray analysis revealed no common upregulation of GPCR, including G_s_, G_i_, or G_q_, involved in salivary secretion under the lunar gravity environment in MHU-4 and -5 missions. **(B)** There were no significant upregulation of autonomic nerve related receptor genes, including adrenergic and cholinergic receptors. Red in heat map indicates upregulated mRNA at 1/6 *g* and green in heat map indicates downregulated mRNA at 1/6 *g* compared to the 1 *g* group.

We explored upstream signaling pathways, including G_s_, G_i_, or G_q_ protein-coupled receptors and their respective second messengers and enzyme-related factors, as well as mRNA related to adrenergic receptors in the sympathetic nervous system and acetylcholine receptors in the parasympathetic nervous system. We observed no significant differences in mRNA expression of them between the samples of GC and PG in both the MHU-4 and MHU-5 missions (Figure 8A). In sympathetic and parasympathetic nerve functions, genes related to adrenergic and cholinergic receptors in the submandibular glands were not significantly upregulated in 1/6 *g* lunar gravity in both the MHU-4 and -5 groups (Figure 8B). Therefore, changes in mRNA expression may occur downstream signals of salivary secretion system involved in vesicular transport and secretion at apical side of acinar cells.

**FIGURE 9:**
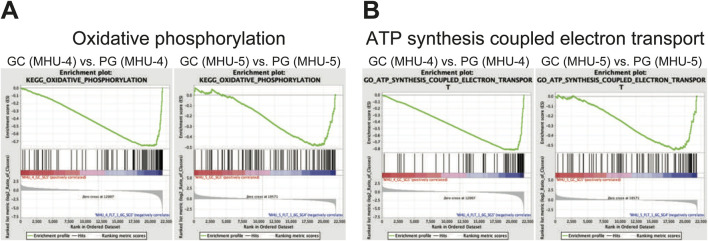
Enrichment in a set of mRNAs relating oxidative phosphorylation and ATP synthesis coupled electron transport was observed in lunar gravity in both MHU-4 and -5 missions. **(A)** Gene set enriched analysis (GSEA) revealed the activation of oxidative phosphorylation in lunar gravity for both MHU-4 and -5 missions. **(B)** GSEA also revealed the activation of ATP synthesis coupled electron transport in lunar gravity for both MHU-4 and -5 missions.

GSEA results revealed that the gene set involved in oxidative phosphorylation were highly upregulated in submandibular gland samples under 1/6 *g* lunar gravity in MHU-4 and -5 (Figure 9A). In addition, the gene set involved in ion exchange associated with ATP synthesis was also upregulated at 1/6 *g* lunar gravity compared to that under 1 *g* in the MHU-4 and -5 missions (Figure 9B).

**FIGURE 10:**
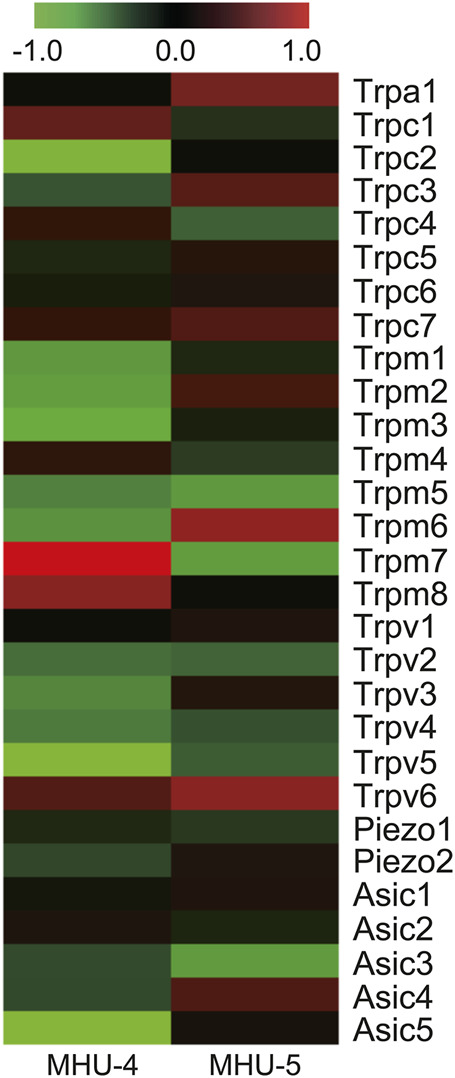
Downregulation of mechanosensitive ion channels in lunar gravity. Heat map showing the ratio calculated from the signal intensity of 1/6 *g*–1 *g*. Microarray analysis revealed downregulation of mechanosensitive ion channels, such as *Trpv2*, *Trpv4*, *Piezo1*, *Piezo2*, and *Asic3*, in lunar gravity for both the MHU-4 and MHU-5 missions. Red in heat map indicates upregulated mRNA at 1/6 *g* and green in heat map indicates downregulated mRNA at 1/6 *g* compared to the 1 *g* group.

We then investigated whether mechanosensitive ion channels in the submandibular glands sense gravity-induced fluid changes. The heat map shows ratio in changes in the mRNA expression of cation channels, especially transient receptor potential (TRP), Piezo, and acid-sensing ion (ASIC) channels. We revealed mechanosensitive ion channel genes, such as *Trpv2*, *Trpv4*, *Piezo1*, *Piezo2*, and *Asic3* were downregulated in the 1/6 *g* group compared with the 1 *g* group in MHU-4 and -5 (Figure 10).

## 4 Discussion

In the 1/6 *g* lunar gravitational environment, *Amy1* was upregulated in the submandibular glands, as were *Vamp8* and the small G protein *Rap1* (*Ras* family)/*Rab2a*, *Rab10*, and *Rab27* (Rab family) which are involved in vesicle secretion. Amylase has been identified as a stress marker in behavioral medicine and space flight crews (Ali and Nater, 2020; Ghasemi et al., 2021). *Vamp8* acts as a v-SNARE that regulates the secretion of the entire exocrine system (Wang et al., 2007). Salivary proteins are synthesized in acinar cells. Based on the genetic information in acinar cells, salivary proteins are synthesized in ribosomes, pass through the rough endoplasmic reticulum, become vesicles, and are transported to the Golgi apparatus. Intravesicular proteins that undergo modifications, such as glycosylation, in the Golgi apparatus are concentrated and stored in secretory granules. Small G proteins such as Ras and Rab families function salivary secretion, as guanosine triphosphate (GTP)-binding proteins with a molecular weight of 20–30 kDa. This hydrolysis results in inactive guanosine diphosphate (GDP). Small G proteins act as molecular switches that bind specific target molecules and transmit intracellular signals. GDP/GTP exchange proteins (GEF) are regulated by GTPase-activating proteins (GAPs). The combined GTP and GDP states function as molecular switches corresponding to the on/off states. Activity regulation is temporally and spatially controlled and functions as a biotimer. Small G proteins are classified into five families, and regulate various cellular functions. *Rap*/*Rab* signaling plays an important role in saliva and amylase secretion (D'Silva et al., 1998; Sabbatini et al., 2008; Gomi et al., 2017). Our study showed no increase in the expression of mRNA related to receptors for noradrenaline secreted from sympathetic nerves involved in protein secretion, where is the upstream of salivary secretion system, while upregulations of Rap/Rab signaling for transporting intracellular vesicles and v-SNARE (Vamp8) to secrete amylase via exocytosis at apical side of acinar cells were observed in the 1/6 *g* environment.

In addition, salivary secretion requires high levels of adenosine triphosphate (ATP) not only for salivary secretion by maintaining intracellular K^+^ concentration in acinar cells, but also for modification of saliva by reabsorption and secretion in ductal cells of submandibular gland. Our GSEA data show that a set of mRNAs involved in oxidative phosphorylation was highly upregulated under 1/6 *g* lunar gravity in MHU-4 and -5. Plus, mRNAs in association of ionic transport coupled with ATP synthesis or hydrolysis were also upregulated at 1/6 *g* lunar gravity compared to that under 1 *g* in the MHU-4 and -5 missions. We also hypothesized that changes in gravity may induce changes in body fluids, resulting in excessive fluid inflow into the submandibular gland. Our data shows downregulation of mechanosensitive ion channels such as *TRP*, *Piezo*, and *ASIC* channels, however. Fluid balance fluctuates when gravity changes, shifting fluid to the upper body and creating a situation where the astronauts have edema around the salivary glands, which causes mechanical stress. It is possible that the mRNA levels of these mechanosensitive ion channels in the submandibular gland decrease because of a function that restores them to their normal state.

**FIGURE 11:**
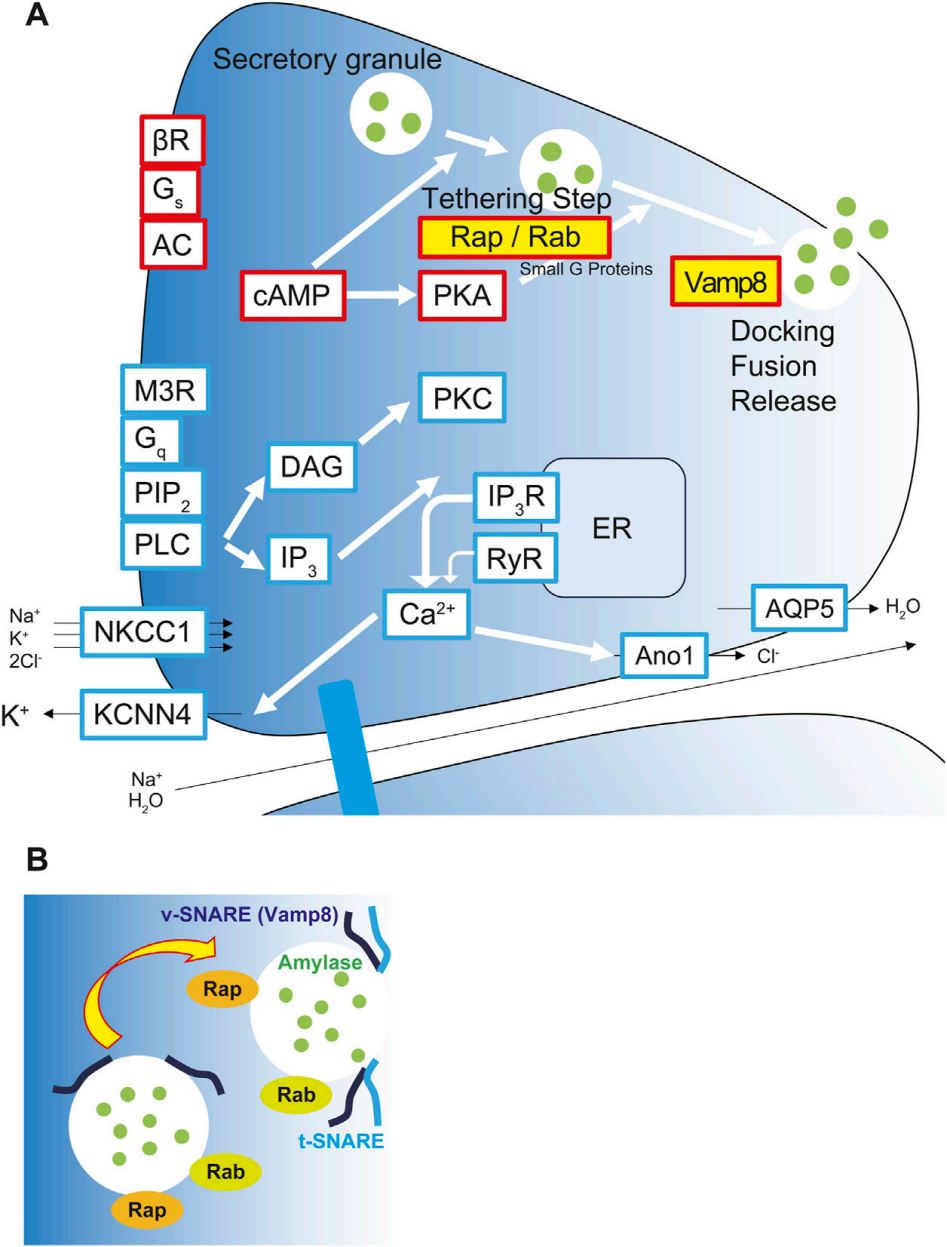
Upregulation of salivary amylase secretion via Rap/Rab signaling and exocytosis via Vamp8 under the lunar gravity. **(A)** Graphical scheme shows signaling pathway for salivary secretion in acinar cells. Yellow labeled Rap-, Rab-, and Vamp8-encoding mRNAs and their proteins were upregulated and localized at apical membrane in acinar cells at the 1/6 *g* lunar gravity. Red boxes represent proteins and molecules for salivary protein secretion. Blue boxes represent proteins and molecules for the water/ion secretion. **(B)** Rap and Rab small G proteins modulate the processivity of vesicles to apical side of cell membrane. Apical side of cell membrane showed upregulation of Vamp8 protein and vesicle-membrane docking to secrete salivary amylase at 1/6 *g* environment. AC, adenylyl cyclase; Ano1, anoctamin1; AQP5, aquaporin 5; βR, β-adrenergic receptor; cAMP, cyclic adenosine monophosphate; Ca^2+^, calcium ion; DAG, diacylglycerol; ER, endoplasmic reticulum; G_q_, G_q_ protein-coupled receptor; G_s_, G_s_ protein-coupled receptor; IP_3_, inositol trisphosphate; IP_3_R, inositol trisphosphate receptor; KCNN4, potassium calcium-activated channel subfamily N member 4; M3R, muscarinic acetylcholine receptor M_3_; NKCC1, sodium, potassium, chloride cotransporter 1; PIP_2_, phosphatidylinositol 4,5-bisphosphate; PKA, protein kinase A; PKC, protein kinase C; PLC, phospholipase C; Rab, small G protein Rab; Rap, small G protein Rap; RyR, ryanodine receptor; t-SNARE, target membrane-associated T-soluble *N*-ethylmaleimide-sensitive factor attachment protein receptor; Vamp8, vesicle-associated membrane protein 8; v-SNARE, vesicle-associated V-soluble *N*-ethylmaleimide-sensitive factor attachment protein receptor.

In conclusion, our results indicate that changes in the gravitational environment induce qualitative changes in salivary secretion function and that multiple genes may be involved in maintaining salivary secretion homeostasis in the space environment. Overall, our data suggested that lunar gravity upregulates salivary amylase secretion via vesicular transport and exocytosis mediated by Rap/Rab signaling and Vamp8 at apical side of acinar cells (Figure 11). However, we have not shown the functional assay as amylase secretion in this study. To clarify the correlation mechanisms between these mRNAs and amylase secretion, it will need to be verified whether any effect on amylase secretion is caused by both gain-of-function and loss-of-function experiments in acinar cells. Further research on salivary secretion signal and gene regulation may contribute to the future monitoring of the physical and mental health of humans living in space as well as the improvement of oral medicine.

## Data Availability

The original contributions presented in the study are included in the article/Supplementary Material, further inquiries can be directed to the corresponding author.
